# Solvent Extraction of Polyphenolics from the Indigenous African Fruit *Ximenia caffra* and Characterization by LC-HRMS

**DOI:** 10.3390/antiox7080103

**Published:** 2018-08-01

**Authors:** Dewald Oosthuizen, Neill J. Goosen, Maria A. Stander, Aliyu D. Ibrahim, Mary-Magdalene Pedavoah, Grace O. Usman, Taiwo Aderinola

**Affiliations:** 1 Department of Process Engineering, Stellenbosch University, Stellenbosch 7600, South Africa; oosthuizen.dewald@gmail.com; 2 Central Analytical Facility, Stellenbosch University, Stellenbosch 7600, South Africa; lcms@sun.ac.za; 3 Department of Microbiology, Usmanu Danfodiyo University, Sokoto PMB 2346, Nigeria; aid4life@yahoo.com; 4 Department of Applied Chemistry and Biochemistry, University for Development Studies, Navrongo, Ghana; mmpeddy@yahoo.com; 5 Department of Food, Nutrition and Home Sciences, Kogi State University, Anyigba 1008, Nigeria; ojaligu@yahoo.co.uk; 6 Department of Food Science and Technology, The Federal University of Technology, Akure PMB 704, Nigeria; aderinolata@futa.edu.ng

**Keywords:** bioactive phytochemicals, flavonoids, indigenous African fruit, natural antioxidants, polyphenols, solvent extraction

## Abstract

Indigenous and non-commercial fruits can be an important source of antioxidant polyphenols; however, the identity and content of polyphenols from non-commercial fruits are often poorly described. The study aimed to extract, identify, and quantify polyphenols from the skin of the indigenous Africa fruit *Ximenia caffra*, using solvent extraction. Three solvents (hexane, acetone, and 70% *v*/*v* ethanol) over three extraction times (30, 60 and 120 min) were used in a 3^2^ full factorial experimental design to determine effects on polyphenol recovery, and individual polyphenolics were characterised using liquid chromatography high-resolution mass spectrometry (LC-HRMS)*.* Ethanol was the most effective extraction solvent, and extracts had high levels of total phenolics and flavonoids (65 mg gallic and 40 mg catechin equivalents per gram dry sample respectively), and high antioxidant activity (18.2 mg mL^−1^ ascorbic acid equivalents). LC-HRMS positively identified 16 compounds, of which 14 were flavonoids including flavonoid glycosides, and indicated that concentrations of some flavonoids decreased for extraction times beyond 60 min. It was concluded that the fruit of *Ximenia caffra* is rich in natural polyphenolic antioxidants; the present work identified and quantified a number of these, while also establishing suitable solvent extraction conditions for the recovery of these potentially high-value compounds.

## 1. Introduction

Fruits of the indigenous African tree *Ximenia caffra* (Sonder) have been shown to contain high amounts of polyphenolic compounds in both the pulp and skins [1]. Natural polyphenols are known for their antioxidant properties, and the evidence linking regular consumption of these natural antioxidants with improved health outcomes is continually growing [2,3,4]. *Ximenia caffra* has been identified as a promising indigenous African plant for commercialisation due to its variety of commercial, cultural, and medicinal uses [5,6,7]; it is widely distributed within and indigenous to Southern and Eastern Africa, including Madagascar [6]. In season, the plant produces edible fruits which are utilised as food by humans and animals, while different parts of the plant including the leaves, bark, and roots are used in traditional medicine to treat a range of diseases and disorders [6,8,9,10], with reports of antibacterial and antifungal activity of *X. caffra* extracts providing support for the ethnomedicinal uses of the plant [9,11]. Commercial activity around the fruit has thus far mainly revolved around production of jellies and jams, and extraction of the commercially valuable seed oil [6,12], and very little attention has been devoted to studying the high amount of polyphenols in the fruit.

The fruit pulp and skins of *X. caffra* are a potential source from which natural antioxidants could be extracted, especially the skins, which contain higher amounts of polyphenols than the pulp, and which are regularly not consumed due to poor palatability [1,6,13]. Polyphenols from plants are of particular interest due to their potential health benefits, disease prevention, and pharmacological activity [2,14,15,16]; natural polyphenols are particularly sought after for their antioxidant activity, especially in the large and lucrative food preservative market [2,17,18]. Polyphenols can also find applications as food colourants, and as the raw materials for industrial applications e.g., for the production of paints, cosmetics, and paper [16,19].

Little is known about the specific polyphenols that occur in *X. caffra* fruit, or the extraction methods and conditions which would maximise polyphenol recovery. Extraction conditions like the type of extraction process employed, the physical condition of raw material, the extraction conditions, and type of solvent all affect the efficiency of product recovery from plant material [2,16,20,21,22], and therefore need to be considered. Different extraction techniques have been demonstrated for polyphenol extraction, including various non-conventional extraction technologies that may improve extraction efficiency and decrease extraction times [23]; yet, organic solvent extraction and supercritical fluid extraction are the most commonly employed commercial extraction techniques [16,20]. Due to the varying polarities of individual polyphenols, each raw material needs to be specifically matched with the most suitable solvent, and might require the use of combinations of solvents, sequential extraction steps, or the use of co-solvents in the case of supercritical fluid extraction, in order to affect optimal extraction efficiency [16,20,24]. A further complication of polyphenol extraction is that severe extraction conditions e.g., elevated temperature or long extraction times could cause polyphenols to degrade or be converted into undesirable by-products [21,25], thereby further emphasizing the need for determining optimal polyphenol extraction conditions.

The study aimed to optimize extraction conditions for the recovery of polyphenols from the fruit of *X. caffra*, to identify specific compounds in the extracts. This was achieved by employing different extraction solvents and extraction times via Soxhlet extraction, and measuring total phenolic and flavonoid contents, as well as antioxidant activity of the extracts, and identifying individual polyphenols using LC-HRMS.

## 2. Materials and Methods

### 2.1. Chemicals and Reagents

Extraction solvents were purchased from Kimix Chemical and Lab Supplies, Cape Town South Africa, and had the following purities: hexane 96%, ethanol 99.7%, and acetone 99.0% minimum. Folin-Ciocalteu’s phenol reagent and sodium hydroxide were purchased from Merck (Darmstadt, Germany). Sodium carbonate, gallic acid, ±catechin hydrate, sodium nitrite, *N*,*N*-Dimethyl-*p*-phenylenediamine dihydrochloride (DMPD), iron(iii)chloride, l-ascorbic acid, sodium acetate trihydrate, aluminium chloride and 0.1 M acetic acid were purchased from Sigma-Aldrich (St. Louis, MO, USA). All chemicals were of analytical grade. Phenolic and flavonoid standards were obtained from Sigma (Darmstadt, Germany).

For all high performance liquid chromatography (HPLC) analyses, methanol and acetonitrile were purchased from Merck Millipore (Darmstadt, Germany) and Sigma-Aldrich (St. Louis, MO, USA), respectively, while HPLC-grade water was prepared using tandem Elix and Milli-Q academic (Merck Millipore) water purification systems.

### 2.2. Raw Material Collection and Preparation

Ripe fruits of *X. caffra* var. *natalensis* were collected in the Hoedspruit area, Limpopo Province, South Africa on the 10 January 2015, at the GPS coordinates: 24°25′12″ S 30°47′42″ E. Fruits were positively identified by a local botanist, Mr. Dave Rushworth, and were kept on ice until processed. Processing entailed separating the skin and fleshy portion of the fruit from the seeds through physical means, homogenizing the fruit flesh and skins together in a food processor and filtering the resultant pulp through a cotton cloth. The filtered juice was frozen at −20 °C, and the solid residue remaining after filtration was then dried in a drying oven at 55 °C for approximately 6 h, and crushed by hand using a mortar and pestle until all material passed a mesh size of 2 mm. The dried material was thoroughly mixed by hand in order to ensure homogenous distribution, and stored in airtight containers at room temperature until used for solvent extraction. The juice was utilised for further nutritional evaluation, for which the findings will be reported elsewhere.

### 2.3. Experimental Design and Extraction Equipment

A 3^2^ full factorial design with 5 replicates was employed to investigate the effects of two factors (type of solvent, and extraction time) each at three different levels, on the extraction of polyphenols from the raw material using the Soxhlet extraction method. As certain polyphenolic compounds can degrade when subjected to elevated temperatures for extended times [26], the appropriate extraction time for polyphenolic recovery needs to be determined. The solvents were chosen to represent three different polarities: hexane (non-polar), acetone (intermediate polarity), and a 70 vol % aqueous ethanol solution (polar, although slightly less polar than water, as suggested by Kim and Lee [27]). The extraction times chosen were 30, 60, and 120 min. The solvent substrate ratio was chosen as 10:1 (volume per weight) as per Aspé and Fernández [26], and kept constant for each run.

At the end of the trial, a single extraction using 70% ethanol as a solvent was also done to generate samples within which individual phenolic compounds were identified; experimental conditions and sampling procedures were identical to the other ethanol extraction runs, and samples were taken at 30, 60 and 120 min extraction time.

The extraction apparatus consisted of a standard Soxhlet setup, with glass thimble holder (150 mL), condenser, and a three-necked 500 mL round bottom solvent flask. The heating source used was an adjustable heating mantle (MRC Scientific Instruments model MNS-500, Holon, Israel).

### 2.4. Extraction and Sampling Procedures

To perform the extraction, 20 g of raw material was loaded into a cellulose extraction thimble (Whatman 603, diameter of 33 mm and length of 100 mm), 200 mL of the solvent was loaded into the round bottom solvent flask, and the extraction was run under full reflux conditions at a constant heating rate. Extraction times were measured from the first syphoning of the solvent. Samples of 5 mL each were taken at 30, 60, and 120 min directly from the round bottom solvent flask, the solvent was removed through evaporation in a vacuum oven, and samples were stored in airtight containers at 4 °C until analysis. Solvent boiling temperature was monitored using a thermocouple inserted through one of the necks of the round bottom solvent flask and recorded every 10 min, and the number of syphoning cycles during the extraction run was recorded.

### 2.5. Analytical Methods

Before sample analysis, stored samples were reconstituted in 5 mL demineralised water. All extraction samples were assayed for total phenolics and total flavonoids, and the ethanol extracts were further assayed for antioxidant activityaccording to the *N*,*N*-Dimethyl-*p*-phenylenediamine dihydrochloride (DMPD) assay.

#### 2.5.1. Total Phenolic Assay

Total phenolics were determined according to the spectrophotometric Folin-Ciocalteu method of Singleton and Rossi [28], with slight modifications. Gallic acid was used as standard, and all values were expressed as gallic acid equivalents (GAE). Sample or standard solutions of 200 μL were added to a 4 mL cuvette, followed by 1000 μL of Folin-Ciocalteu reagent. The mixture was incubated for 8 min at room temperature and in the dark, after which 800 μL of a 7.5% sodium carbonate solution was added. A final amount of 1000 μL of demineralised water was then added, and the absorbance of the sample was measured with a spectrophotometer (A&E Lab, United Kingdom, model AE-S60-4U) at 765 nm. Blanks were prepared using demineralised water.

#### 2.5.2. Total Flavonoid Assay

Total flavonoids were measured using the method described by Amado, et al. [29], with catechin as standard, and values were expressed as catechin equivalents (CE). A volume of 1000 μL of sample or standard was pipetted into a 4 mL cuvette, and 75 μL of 5% NaNO_2_ solution was added and incubated at room temperature. After six minutes’ incubation, 150 μL of 10% AlCl_3_ was added to the solution and incubated for a further 5 min, after which 500 μL of 1 M NaOH was added. The solution was then made up to 2.5 mL by the addition of 775 μL of demineralised water, and incubated for 30 min before measuring the absorbance at a wavelength of 410 nm. Blanks were prepared using demineralised water.

#### 2.5.3. DMPD Antioxidant Activity Assay

Only the 70% ethanol extracts were subjected to evaluation of antioxidant activity, due to difficulties in solubilisation of dried acetone and hexane extracts in the aqueous medium required to perform the antioxidant activity, and because the 70% ethanol extracts showed the highest polyphenolic and flavonoid content. The DMPD assay as described by Fogliano, et al. [30] was used for analysis. DMPD reagent was prepared by mixing 1000 μL of 100 mM DMPD solution, 200 μL of 0.05 M FeCl_3_ solution, and 100 mL of pH 5.25 acetate buffer. This reagent was stored in the dark at 4 °C until use. To perform the assay, 100 μL of sample and 2 mL of the buffered 100 mM DMPD reagent were added to a cuvette, mixed, and incubated for 10 min at ambient temperature. Absorbance was then measured at 505 nm, with ascorbic acid used as standard and demineralised water as blank.

#### 2.5.4. Identification of Phenolic Compounds

A Waters Synapt G2 quadrupole time-of-flight mass spectrometer connected to Waters Ultra pressure liquid chromatograph and photo diode array detection was used for LC-HRMS analysis. A previously published method [31] was used which employs a gradient specifically focusing on phenolic acids and flavonoids. The only difference was that a Waters BEH C18, 2.1 × 100 mm, 1.7 µm column was used. In short, a 0.1% formic acid (solvent A) to acetonitrile containing 0.1% formic acid (solvent B) gradient was applied up to 28% solvent B, followed by a wash step.

The instrument was operated using electrospray ionisation in negative MS^E^ mode, which consisted of a low collision energy scan (6 V) from *m/z* 150 to 1500, and a high collision energy scan from *m/z* 40 to 1500. Positive identification of compounds was based on retention time matching with authentic standards, accurate mass data, ultraviolet (UV) data, as well as mass spectrometry-mass spectrometry (MSMS) fragmentation data.

Ethanol extracts obtained via Soxhlet extraction were diluted five and ten fold in 50% methanol/water, centrifuged, and the supernatant injected directly into the system. A methanol extract was also prepared from the same raw material as used for Soxhlet extraction, and characterized for polyphenolic content. A cocktail of the standards were injected unto the system at (100, 50, 25, 10, 5 and 0.5 mg L^−1^), and the application manager Targetlynx 4.1 (Waters, Milford, MA, USA) was used for the quantifications.

### 2.6. Statistical Analysis

Data on total phenolics, flavonoids, and antioxidant activity were analysed using the Statistica version 12 software package. Data were subjected to analysis of variance (ANOVA) and least significant difference (LSD) tests, using the Variance Estimation and Precision (VEPAC) module in Statistica. Main effects and interaction effects were estimated, and differences were deemed to be significant for *p* < 0.05.

## 3. Results

There were noticeable variations in the final colour of the extraction solvent collected in the round bottom flask between the solvents, with the 70% ethanol having an intense orange-red colour corresponding to that of the original raw material. Acetone extracts showed intermediate colour, while hexane extracts exhibited very little colour change.

**Figure 1:**
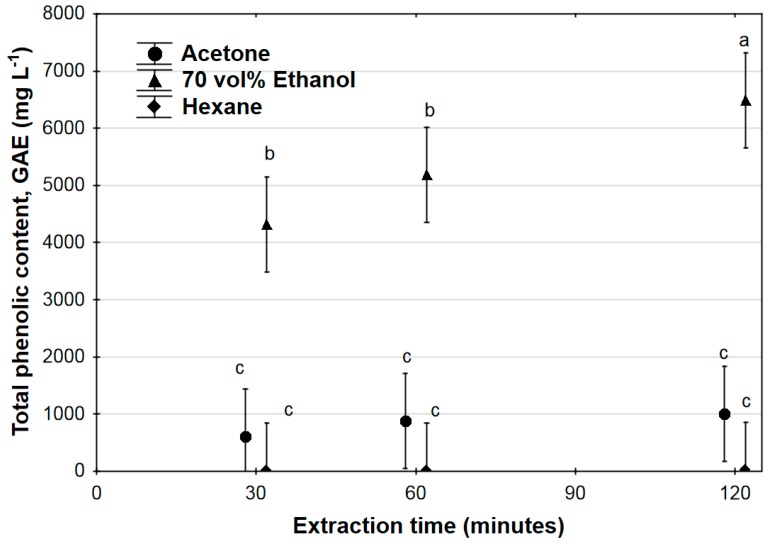
Total phenolic content of *Ximenia caffra* extracts over time, for different solvents, expressed as gallic acid equivalents (GAE). Data are represented as mean ± 95% confidence intervals. Values with common superscripts do not differ significantly.

Figure 1 shows the extraction of total phenolics over the course of 120 min, for all three solvents. Total phenolic content was significantly higher (*p* < 0.05) in the 70% ethanol extract than for the other solvents, at all sampling times during the extraction. Within the 70% ethanol extraction, total phenolics extracted increased significantly over time from 4312 ± 1819 mg L^−1^ GAE (mean ± SD) at 30 min to 6487 ± 1203 mg L^−1^ GAE at 120 min, but there was no significant difference between phenolics after 30 min and 60 min extraction time. No significant differences in total phenolics concentration were found between hexane and acetone extracts, and within each of these two solvents, the total phenolic concentration did not vary significantly between 30 min and 120 min extraction time. The interaction between solvent and time was found to be statistically insignificant for all solvents (*p* > 0.05).

**Figure 2:**
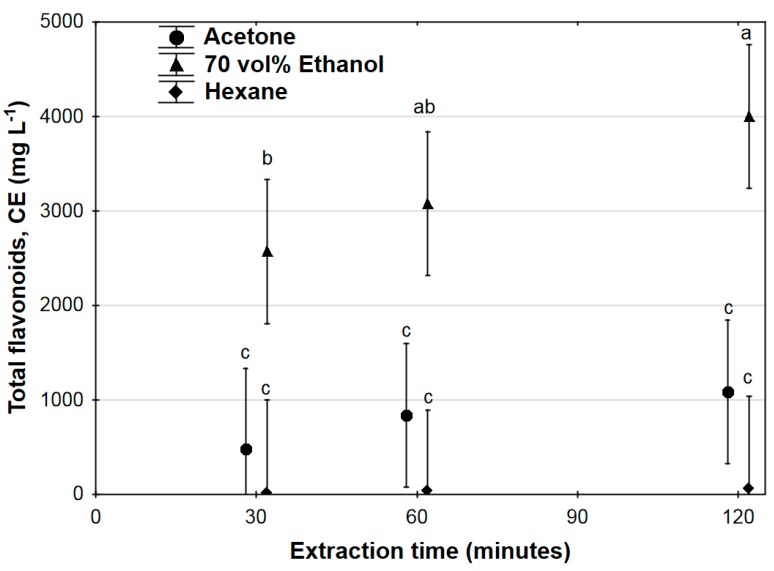
Total flavonoid content of *Ximenia caffra* extracts over time, expressed as catechin equivalents (CE). Data are represented as mean ± 95% confidence intervals. Values with common superscripts do not differ significantly.

Extraction of total flavonoids over time for all solvents is shown in Figure 2. Total flavonoid extraction was significantly higher for the 70% ethanol extraction than either acetone or hexane at all sampling times, and within the 70% ethanol, total flavonoid concentration increased significantly from 2571 ± 965 mg L^−1^ CE at 30 min, to 4000 ± 1480 mg L^−1^ CE at 120 min extraction time. There were no significant differences in flavonoid content between acetone and hexane extracts, and within each solvent type there were no significant increases in flavonoid concentration between 30 min and 120 min extraction time. No significant time-solvent interaction effect was found for flavonoid extraction.

**Figure 3:**
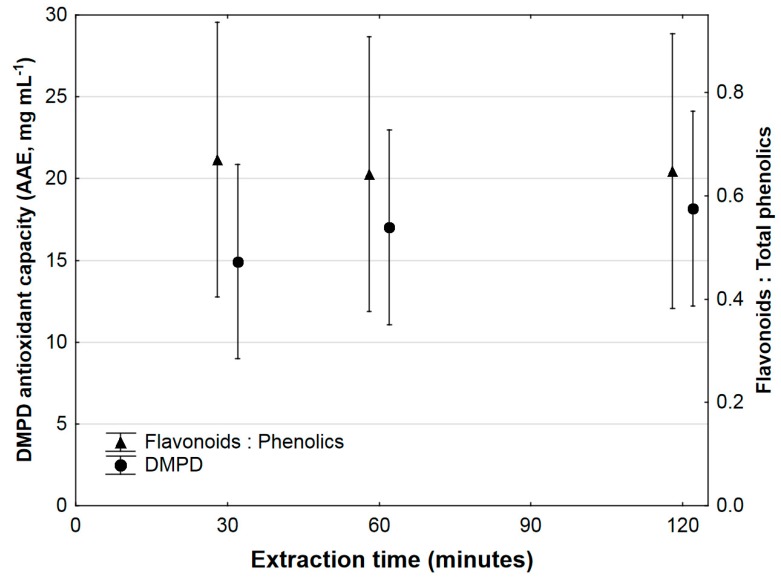
*N*,*N*-Dimethyl-*p*-phenylenediamine dihydrochloride (DMPD) antioxidant capacity and ratio of total flavonoids to total phenolics, for 70% ethanol extracts of *Ximenia caffra*. Data are represented as mean ± 95% confidence intervals.

Figure 3 shows the results of DMPD antioxidant activity, and the ratio of flavonoids to phenolics, for the 70% ethanol extracts between 30 min and 120 min extraction time. There was a numerical increase in antioxidant activity from 14.9 mg L^−1^ AAE at 30 min to 18.2 mg L^−1^ AAE at 120 min; however, the increase was not statistically significant. Within the 70% ethanol solvent, the mean values for the ratio of total flavonoids to total phenolics did not differ significantly over time, with a ratio 0.67 being obtained at 30 min, and a value of 0.66 at both 60 and 120 min.

Table 1 lists positively identified compounds obtained during extraction, along with concentrations of these compounds as extracted at different times using a 70% ethanol Soxhlet extraction run. Table 1 further indicates the retention times and other data used to identify individual compounds. The following compounds were positively identified: catechin, citric acid, epicatechin, gallic acid, hesperetin, hyperoside, isoquercitrin, kaemferol glucoside, luteolin-7-*O*-glucoside, procyanidin B1, procyanidin B2, quercetin-3-*O*-glucoside, quercetin-3-*O*-robinobioside, quercetin, rutin, and trilobatin. A number of compounds were tentatively identified, or could not be identified; concentrations of these compounds in the solvents were not determined. Please refer to Figure S1 for a chromatogram showing retention times and peaks of the positively identified compounds.

Citric acid was the dominant compound extracted in all ethanol extractions, with a highest concentration of 1313 mg L^−1^ at 60 min extraction time, and a final concentration of 1288 mg L^−1^ at 120 min, followed by procyanidin B1 (60.0 mg L^−1^), cathechin (54.9 mg L^−1^), isoquercitrin (50.8 mg L^−1^) and hyperoside (40.8 mg L^−1^) after 120 min extraction time. For all compounds except quercetin-3-*O*-robinobioside, extracted concentrations increased from 30 min to 60 min extraction time. However, for hyperoside, rutin, quercitin, quercitin-3-*O*-glycoside, isoquercitrin and citric acid, the extracted concentrations decreased between 60 min and 120 min extraction time, while for all other compounds, the concentrations increased or remained constant.

## 4. Discussion

The current study reports findings on solvent extraction of polyphenols displaying high antioxidant activity from the fruits of *X. caffra*, and reports the identity of the individual compounds obtained in the ethanol extracts. There has recently been increased interest in the commercialisation of *X. caffra*, due to the range of different potential products which can be derived from this versatile plant species [6]; however, information on the individual compounds responsible for the high antioxidant capacity, and potential processing methods for specific product recovery from the different plant portions is lacking.

Optimal extraction of phenolic compounds and flavonoids, as measured with the spectrophotometric methods, was achieved using 70% aqueous ethanol as extraction solvent at 120 min extraction time. Phenolic acid and flavonoid recovery were both significantly affected by the choice of extraction solvent and length of extraction, while interaction effects between these two parameters were statistically insignificant. The results of the current study are in line with previous work on extraction of polyphenols from plant materials. It is known that the selection of an appropriate solvent is an important processing parameter during polyphenolic extraction, as extraction yield, total phenolic content, and antioxidant capacity of extracts, extraction kinetics and the bioactivity of the final extracts can all be affected by the choice of solvent [19,32,33]. The polarity of extraction solvents has been proposed to be one of the critical aspects resulting in differences in extraction efficiency [32,34]. The current study found large differences in extraction of polyphenol between the solvents, with the less-polar solvents hexane and acetone resulting in poor phenolics extraction. Previous work on *X. caffra* utilised methanol for extraction of phenolic compounds [1,35]; however, for the current study, ethanol was preferred, due to its decreased toxicity compared to methanol.

The significant effect of extraction time on the extraction of phenolics and flavonoids when using 70% ethanol as the solvent is apparent from Figure 1 and Figure 2, where the optimal extraction time (based on spectrophotometric determination of total phenolics and total flavonoids) was found to be 120 min in both instances, with significant increases occurring throughout the entire extraction run. Time dependence of phenolic acids and flavonoid extraction was not found for either acetone or hexane as solvents; this is ascribed to the overall low extraction achieved using these solvents. However, despite longer extraction times indicating higher overall polyphenolic extraction, the data in Table 1 clearly indicates decreases in concentration of certain compounds between 60 and 120 min extraction time. These observed decreases in concentration may be due to the fact that some polyphenolics can degrade over time when kept at elevated temperatures [36,37,38,39,40], and it is clear from the data that compounds differed in this regard, e.g., the extracted concentrations of catechin and procyanidin B1 increased from 60 to 120 min extraction time, while extracted concentrations for hyperoside and isoquercitrin decreased over the same time period. Although further phenolic acid and/or flavonoid recovery may be achieved by extending extraction time beyond 120 min, this needs to be balanced against the potential time-dependent degradation of thermally labile components in the extracts [26].

The fruit of *X. caffra* are a good overall source of phenolic compounds. Relatively high amounts of total phenolics and flavonoids were extracted by the 70% ethanol solution from the raw material after 120 min of extraction time, corresponding to 6487 ± 1203 mg L^−1^ GAE and 4000 ± 1480 mg L^−1^ CE respectively in the extracts, which translates to 65 mg g^−1^ GAE dry sample weight for total phenolics, and 40 mg g^−1^ CE dry sample weight for total flavonoids. By way of comparison, total phenolics extracted from blueberries, a fruit generally acknowledged to be high in antioxidants and phenolics, amounted to a maximum of 9.44 ± 0.22 mg g^−1^ GAE per dry weight [41], and 6.94 ± 0.47 mg g^−1^ GAE per gram fresh fruit weight [42] in two different studies. *X. caffra* fruit pulp and peels have been reported to have high antioxidant capacity, and to contain high total phenolic and proanthocyanidin levels [1,35]; the findings in the current study are therefore in agreement with those of prior studies. The major individual polyphenolic compounds identified within the extracts were catechin, hyperoside, isoquercitrin, and procyanidin B1 (all at levels higher than 40 μg mL^−1^ after 120 min extraction time). Both hyperoside and procyanidin B1 are known as strong antioxidants [43,44], while catechin is associated with improved cardiovascular health [45]. Isoquercitrin is a glycoside of quercetin and has been demonstrated to exhibit a number of potentially beneficial biological effects linked to its ability to act as antioxidant [46].

The majority of the polyphenols recovered using Soxhlet extraction consisted of flavonoids. Among all the compounds which could be positively identified with LC-HRMS, only citric acid and gallic acid are neither flavonoids nor glycosides of flavonoids (see Table 1). Citric acid is not a polyphenol, but is retained in the data as it can act as antioxidant in food systems, and it contributes to the antioxidant activity measured using the DMPD method [47,48]. The ratio of total flavonoids to total phenolics seemed to remain fairly constant when calculated based on spectrophotometric analyses, and remained between 0.66 and 0.67 over the whole extraction time (Figure 3). This apparent constant ratio of total flavonoids to total phenolics indicates that there is no preferential extraction of flavonoids relative to other phenolic compounds, or that the proportion of non-flavonoid phenolics recovered are much lower relative to flavonoids, and therefore, any potential differences cannot be determined from spectrophotometric analyses. One potential practical implication of this is that optical process monitoring can be employed during extraction, as increased phenolic extraction corresponded with increased colour in the extraction solvent. As flavonoids particularly contribute to colour in fruit [49], the monitoring of extract colour can provide a rapid method for determination of approximate flavonoid concentration in extracts.

The DMPD antioxidant activity did not correspond to the increased extraction of total phenolics and flavonoids over time in this investigation. Even though Soxhlet extraction lead to a numerical increase in DMPD antioxidant activity from 30 to 120 min extraction, the increase was not statistically significant. Two contributing factors for this could be degradation of certain phenolic compounds in the extracts (and therefore decreased antioxidant activity), as seen in the data of Table 1, and the possibility that the majority of compounds that exhibit antioxidant activity were recovered within the first 30 min of the extraction. The data in Table 1 indeed confirm that, for all compounds which were positively identified, the majority of phenolics’ extraction took place during the first 30 min. Additionally, it is also possible that the total phenolic content in the extract was not a good predictor of total antioxidant activity in this instance. Phenolics are known to be a very diverse group of plant compounds [2], and different phenolic compounds have different reactions with regards to total phenolic and antioxidant assays [16,50,51,52]. Further, phenolic compounds are not the only compounds in plant extracts that contribute toward total antioxidant activity; thus, even though there is generally a correlation between total phenolic content and antioxidant activity [53,54], there have been other reports where higher phenolic content in fruit extracts did not necessarily translate to higher antioxidant capacity [54]. All of these factors may have contributed toward the fact that the DMPD assay did not follow the same trends as those of total phenolics or total flavonoids in this investigation.

## 5. Conclusions

The present work has shown that the fruit skins of *X. caffra* contain high levels of total phenolics and flavonoids, and that relatively high levels of these compounds can be extracted using a 70% aqueous ethanol as solvent. Extraction was time-dependent, and prolonged extraction times, i.e., beyond 60 min, lead to decreases in extraction of certain phenolic compounds, presumably due to heat degradation. The majority of extracted phenolics consisted of flavonoids, and individual compounds were successfully identified and quantified using LC-HRMS. This work makes a contribution toward identifying compounds with high antioxidant activity—and with potentially high-value—that can be extracted from *X. caffra*, and in establishing appropriate extraction conditions for doing so. Future studies should focus on elucidating the antioxidant and bioactivity of the extracts.

## Figures and Tables

**Table 1:** LC-HRMS data obtained for the different extracts from *Ximenia caffra*, detailing compound identity and the time-dependent concentration for the ethanol extracts.

Compound Number	Compound Name	*m*/*z*^1^	Retention Time (min)	[M − H]^−^	MS^E^ Fragments ^2^	Individual Compound Concentrations (μg mL^−1^)			
						Ethanol: 30 min	Ethanol: 60 min	Ethanol: 120 min	Methanol Extraction
**Positively identified compounds**									
1	Catechin	289.0713	11.48	C_15_H_13_O_6_	289,125,203,245,151	36.8	51.9	54.9	68.0
2	Citric acid	191.0187	3.12	C_6_H_7_O_7_	191,111,87,173	1042	1313	1288	2133
3	Epicatechin ^3^	289.0698	13.57	C_15_H_13_O_6_	weak	-	-	-	-
4	Gallic acid	169.0129	5.8	C_7_H_5_O_5_	125,169,111	4.8	5.2	5.8	8.5
5	Hesperetin	301.1643	24.49	C_15_H_25_O_6_	weak	0.1	0.1	0.1	0.1
6	Hyperoside	463.0878	17.51	C_21_H_19_O_12_	300,463,271,255	34.6	42.9	40.8	102.8
7	Isoquercitrin	463.0876	17.51	C_21_H_19_O_12_	300,463,271,301,255,	43.2	53.5	50.8	128.2
8	Kaempferol glucoside	447.0938	18.06	C_21_H_19_O_11_	285,169,447	12.1	12.3	16.7	33.1
9	Luteolin-7-*O*-glucoside	447.0935	18.06	C_21_H_19_O_11_	285,284,169,125,447	0.3	0.3	0.3	1.1
10	Procyanidin B1	577.1317	10.68	C_30_H_25_O_12_	289,407,425,577	36.8	52.5	60.0	203.8
11	Procyanidin B2 ^3^	577.1345	12.72	C_30_H_25_O_12_	289,407,425,577	-	-	-	-
12	Quercetin-3-*O*-glucoside	463.0886	17.81	C_21_H_19_O_12_	300,271,463,255,125	2.7	3.4	3.1	9.0
13	Quercetin-3-*O*-robinobioside	609.1432	17.06	C_27_H_29_O_16_	300,609,271,125	3.9	3.7	5.8	15.4
14	Quercetin	301.0353	23.99	C_15_H_9_O_7_	125,169	2.0	2.2	1.8	1.9
15	Rutin	609.1458	17.27	C_27_H_29_O_16_	300,609,271,255	11.5	13.8	13.1	38.3
16	Trilobatin	435.1284	18.75	C_21_H_23_O_10_	315,345	0.5	0.7	0.8	2.1
**Tentatively identified and unknown compounds ^3^**									
	Aconitic acid	173.0089	9.14	C_6_H_5_O_6_	111				
	Dihydroxy hexadecanoic acid	287.2236	24.5	C_16_H_31_O_4_	287				
	*p*-Coumaroylquinic acid	337.0916	11.18	C_16_H_17_O_8_	163,119,191,337				
	Procyanidin	577.1344	10.68	C_30_H_25_O_12_	289,407,425,577				
	Quercetin galloyl glucoside	615.0979	16.63	C_28_H_23_O_16_	300,615,463,255,169				
	Quercetin galloyl glucoside	615.0977	16.94	C_28_H_23_O_16_	300,463,615				
	Quercetin rhamnoside	447.0927	19.63	C_21_H_19_O_11_	300,271,255,447,243				
	Quercetin rhamnoside	447.0927	18.89	C_21_H_19_O_11_	300,271,255,447				
	Quercetin-3-*O*-pentoside	433.0764	18.83	C_20_H_17_O_11_	300,271,255,433,315				
	unknown	340.1035	14.37	C_15_H_18_NO_8_	161,101,85				
	unknown	443.1913	11.37	C_21_H_31_O_10_	443,289,303				
	unknown	515.1246	5.49	C_18_H_27_O_17_	515,111,173				
	unknown	515.1245	5.68	C_18_H_27_O_17_	515,111,173				
	unknown	515.1255	5.83	C_18_H_27_O_17_	515,111,173				
	unknown	515.1253	5.93	C_18_H_27_O_17_	515,111,173				
	unknown	435.107	21.403	C_24_H_19_O_8_	341,189,125,435				
	unknown	219.0506	9.37	C_8_H_11_O_7_	111,219,87				

^1^ The mass accuracy for all compounds was better than 5 ppm; ^2^ Most abundant fragment mentioned first; ^3^ Concentrations of epicatechin, procyanidin B_2_ and all tentatively identified and unknown compounds were not determined.

## Supplementary Materials

The following are available online at http://www.mdpi.com/2076-3921/7/8/103/s1. Figure S1: Chromatogram showing peaks and retention times of the positively identified compounds. The numbers indicated correspond to the numbering of compounds in Table 1.

## List of Compounds

Catechin	PubChem CID: 9064
Citric acid	PubChem CID: 311
Gallic acid	PubChem CID: 370
Hyperoside	PubChem CID: 5281643
Isoquercitrin	PubChem CID: 5280804
Procyanidin B1	PubChem CID: 11250133
Quercetin-3-*O*-glucoside	PubChem CID: 5748594
Quercetin-3-*O*-robinobioside	PubChem CID: 10371536
Rutin	PubChem CID: 5280805
