# Supplementary material for: Solvent Extraction of Polyphenolics from the Indigenous African Fruit Ximenia caffra and Characterization by LC-HRMS
Source: Antioxidants (Basel). 2018 Aug 1;7(8):103. doi: 10.3390/antiox7080103 (PMC6116166; doi:10.3390/antiox7080103)
Supplement: Supplementary file 1 [file antioxidants-07-00103-s001.zip › antioxidants-334607-supplementary.pdf]

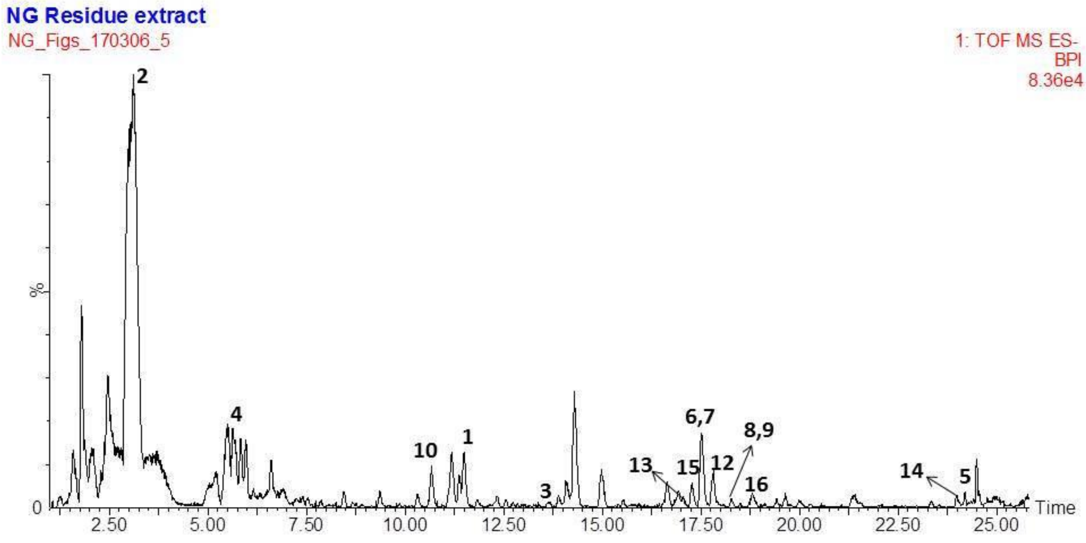

Figure S1. Chromatogram showing peaks and retention times of the positively identified compounds. The numbers indicated correspond to the numbering of compounds in Table 1.
